# Supplementary material for: Predicting visual function by interpreting a neuronal wiring diagram
Source: Nature. 2024 Oct 2;634(8032):113–23. doi: 10.1038/s41586-024-07953-5 (PMC11446822; doi:10.1038/s41586-024-07953-5)
Supplement: Supplementary file 6 — CRF and ERF predictions for individual TmY4 and TmY9 cells. Analogous to Supplementary Data 3, but for TmY target types. Shown are the top four monosynaptic pathways, the strongest pathway passing through each of the top ten intermediary types (ranking from Extended Data Fig. 7), and the trisynaptic pathway Tm1–TmY–Dm3–TmY (see the section entitled Prediction of spatial normalization). [file 41586_2024_7953_MOESM6_ESM.zip › DataS4/TmY9qΓèÑ/720575940619487764.pdf]

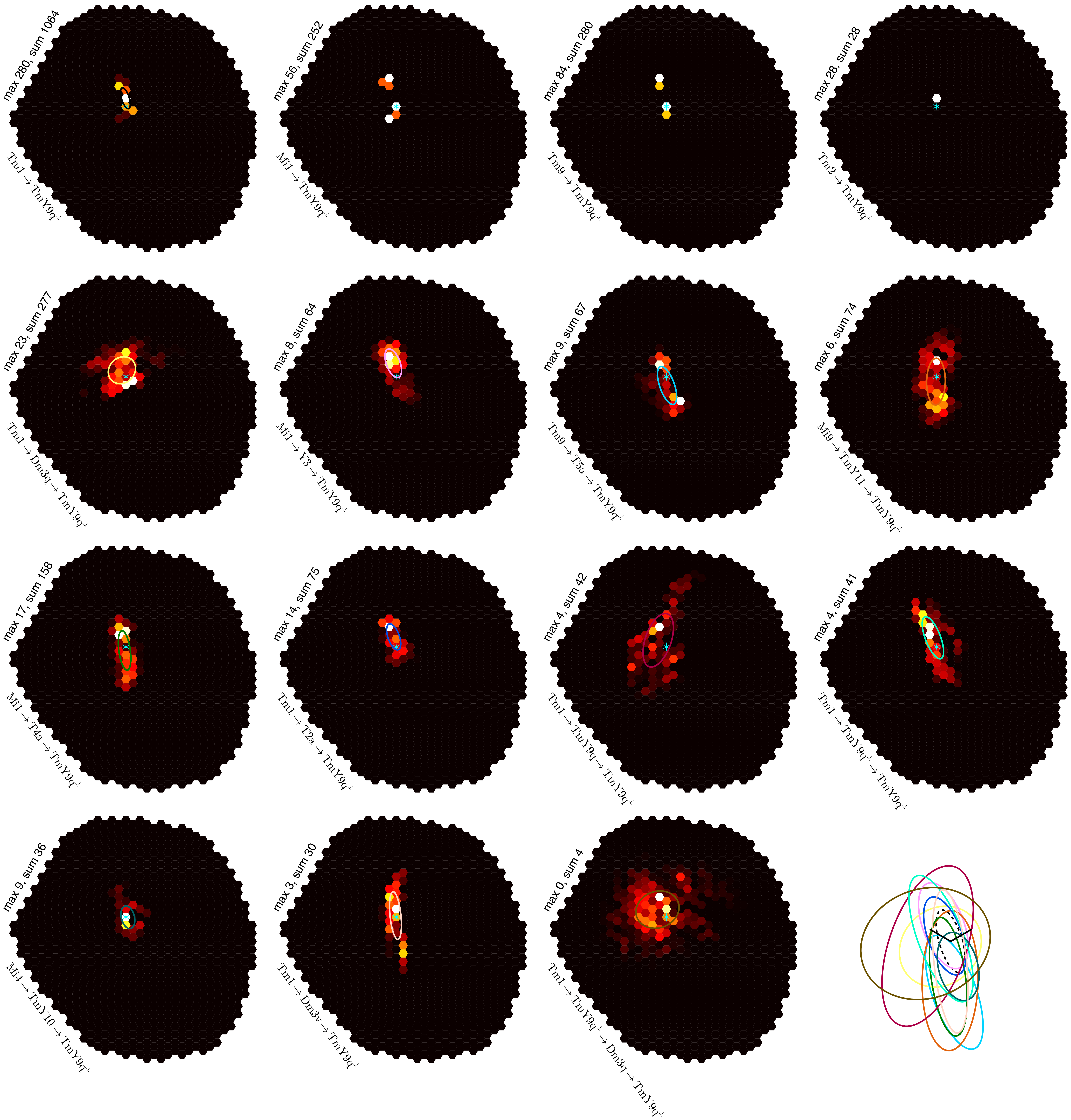
max 280, sum 1064
Tm1 → TmY9q⊥
max 56, sum 252
Mi1 → TmY9q⊥
max 84, sum 280
Tm9 → TmY9q⊥
max 28, sum 28
Tm2 → TmY9q⊥
max 23, sum 277
Tm1 → Dm3q → TmY9q⊥
max 8, sum 64
Mi1 → Y3 → TmY9q⊥
max 9, sum 67
Tm9 → T5a → TmY9q⊥
max 6, sum 74
Mi9 → TmY11 → TmY9q⊥
max 17, sum 158
Mi1 → T4a → TmY9q⊥
max 14, sum 75
Tm1 → T2a → TmY9q⊥
max 4, sum 42
Tm1 → TmY9q → TmY9q⊥
max 4, sum 41
Tm1 → TmY9q⊥ → TmY9q⊥
max 9, sum 36
Mi4 → TmY10 → TmY9q⊥
max 3, sum 30
Tm1 → Dm3v → TmY9q⊥
max 0, sum 4
Tm1 → TmY9q⊥ → Dm3q → TmY9q⊥
